# Barriers to Indigenous Fire Stewardship on Karuk Lands

**DOI:** 10.1002/ece3.73479

**Published:** 2026-04-22

**Authors:** Caitlyn Cruz, Kathy McCovey, Gregory Russell, Dominique David‐Chavez, Lindsey Schneider, Courtney Schultz

**Affiliations:** ^1^ Colorado State University Fort Collins Colorado USA; ^2^ Karuk Tribe/Mid Klamath Watershed Council Orleans California USA

**Keywords:** colonial land management, cultural burning, Indigenous fire stewardship, Karuk tribe, post‐fire rehabilitation

## Abstract

As climate change drives more frequent and intense wildfires, the revitalization of Indigenous fire stewardship grows increasingly urgent. This paper examines the Karuk Tribe's experiences with settler colonialism and their efforts to restore cultural fire stewardship in the wake of the 2020 Slater Fire, which burned 157,000 acres of Karuk ancestral territory. Through a collaborative, community‐engaged case study approach, we conducted 13 interviews with Karuk Tribal members and staff to identify post‐fire recovery priorities, explore management options, and examine governance systems affecting Karuk homelands. Participants emphasized that the criminalization of their traditional fire stewardship practices, now compounded by federal land management practices and ongoing obstacles to restoring their ecocultural stewardship, has resulted in forested landscapes prone to high‐severity fire, posing a threat to their safety and well‐being. Findings highlight how the oppressive forces of settler colonialism persist today, as Karuk people continue to experience barriers to enacting stewardship, sovereignty, and religious freedom. Participants described fire as essential for cultural continuity, ecosystem health, and Karuk governance. The Karuk Tribe's leadership—through policy advocacy, research partnerships, programs like Indigenous Women‐in‐Fire Training Exchange (TREX), and partnerships grounded in Indigenous data sovereignty—offers a model for advancing ecocultural stewardship. Post‐fire landscapes present critical opportunities to reestablish Tribal stewardship and shift to more beneficial fire management paradigms. This research affirms that supporting Indigenous fire knowledge and practice is essential for effective place‐based climate adaptation.

## Graphical abstract

This paper examines the Karuk Tribe's experiences with settler colonialism and their efforts to restore cultural fire stewardship in the wake of the 2020 Slater Fire, which burned 157,000 acres of Karuk ancestral territory. Through a collaborative, community‐engaged case study approach, we conducted 13 interviews with Karuk Tribal members and staff to identify post‐fire recovery priorities, explore management options, and examine governance systems affecting Karuk homelands. Findings highlight how the oppressive forces of settler colonialism persist today, as Karuk people continue to experience barriers to enacting stewardship, sovereignty, and religious freedom.

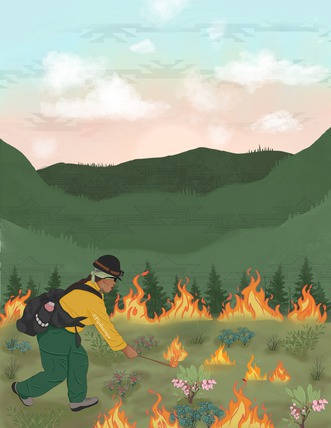

## 1 Introduction

As climate change drives more frequent and intense wildfires (Jones et al. 2022; Armenteras‐Pascual et al. 2025), the revitalization of Indigenous fire stewardship grows increasingly urgent (Whyte 2017). The Karuk Tribe has led efforts to restore cultural burning through policy change (Clark et al. 2021; Clark, Tripp, et al. 2024). As leaders in climate adaptation and science, the Karuk Tribe practices fire stewardship through oral histories, songs, and ceremonies that carry traditional knowledge guiding ecosystem maintenance, supporting conditions for species they depend on, and sustaining their way of life (K. Norgaard 2014a, 2014b; K. M. Norgaard 2019). Colonization disrupted Karuk practices and has led to the development of fire‐prone landscapes that now threaten Karuk lifeways (Curtis 2022). Current exclusion of Indigenous fire stewardship in forest and fire management perpetuates historical injustices, whereas its application is essential for cultural survival, ecological health, and sovereignty (Christianson et al. 2022; Lake et al. 2017).

This paper examines the Karuk Tribe's experiences with settler colonialism and their efforts to reclaim fire stewardship, using the 2020 Slater Fire as a focal point. Post‐fire events create opportunities for dialog and restoration, in part because fires often incinerate commercial tree species, thus reducing land managers' incentives to maintain practices that exclude Indigenous fire stewardship. In the sections that follow, we explore Karuk perspectives on post‐fire management, cultural burning, and pathways to restoring cultural fire.

### 1.1 Indigenous Fire Stewardship

Since time immemorial, Indigenous peoples have worked with fire to steward ancestral lands. Low‐intensity burns improved the abundance and quality of food, fibers, medicines, and cultural resources (Christianson et al. 2022; Goode et al. 2022; Lake et al. 2017). Fire removes underbrush, suppresses pests, stimulates seeds, and attracts wildlife (Connor et al. 2022; Halpern 2016). Indigenous fire stewardship—also called cultural burning—is guided by place‐based knowledge systems developed over generations for survival and cultural continuity (Kimmerer and Lake 2001; A. C. Christianson 2015; Lake et al. 2017; Long and Lake 2018; Whyte 2017; K. M. Norgaard 2019; Roos et al. 2022).

Although fire stewardship methods vary across Indigenous communities, fire is a common element of community, cultural continuity, and land stewardship. Cultural ceremonies, including songs, dances, and regalia, hold ecocultural indicators for Indigenous peoples, guiding when, where, why, and how to carry out land management practices (Kimmerer 2013). These practices create roles in the community to help foster sustainable diets and landscapes that help combat illness through access to nutritional foods and medicinal plants (Lake & A. C. Christianson 2020; Anderson 2006; Roos et al. 2022; Adams 2024).

For the Karuk Tribe, fire is central to ecocultural stewardship and survival. It is used in the Pikyaavish (world renewal) ceremony to manage forests and rivers, and to cultivate grasses and roots for basketry and regalia (Lake et al. 2010). Fire protects communities and resources from high‐intensity wildfires while improving habitat and materials. Smoke shields acorn crops and cools rivers for salmon spawning (K. M. Norgaard 2019). Traditionally, men managed distant hunting grounds while women burned around homes to enhance food and culturally important plants (Curtis 2022). Research suggests Karuk fire stewardship may have entailed as many as 7000 fires annually (Greenler et al. 2024). Indigenous Peoples across the globe continue to recognize fire as a form of medicine, using intentional burning to clear what is unhealthy and promote growth across the landscape (Hoffman et al. 2021). In this way, fire is not only a tool for ecological management but is a means of survival and assertion of Indigenous sovereignty regarding their people, lands, and resources.

### 1.2 Impacts of Colonization on Forest Ecocultural Stewardship

Settler colonialism in North America led to the genocide of Indigenous Peoples. The dispossession, murder, and criminalization of Indigenous people and practices such as fire stewardship, combined with federal land management policies like fire suppression and now compounded by climate change, have increased the risk of high‐severity wildfires nationwide (Fitzpatrick 2024; Clark, Archer, et al. 2024; Maezumi et al. 2024). In the United States, federal policy has long treated land as a financial asset, valued for its ability to generate capital through industries such as mining and timber (Anderson 2006; Norgaard et al. 2011; Curtis 2022). Settler‐colonial notions of land ownership are rooted in the idea of subduing land for resource production, separating nature from society (Kimmerer 2018; Whyte 2017). By contrast, the Karuk worldview recognizes humans as integral to ecosystems, responsible for maintaining balance through active stewardship. Kari Norgaard explains that “Western” constructions of “civilized man” and race classified those not dominating nature as part of it—objects to be subdued like land, rather than people with equal rights (2019). This logic justified genocide, suppression of Indigenous stewardship, and policies undermining sovereignty (K. M. Norgaard 2019; Dunbar‐Ortiz 2023).

Following the violent removal of Native people, the federal government expanded control over Indigenous lands through forest reserves, starting in 1891, which eventually became national forests. For the Karuk Tribe, this included the designation of Klamath National Forest in 1905 and its expansion in 1907, followed by Six Rivers National Forest in 1947 (Quinn 2015). These designations placed most of the Karuk's ancestral lands under federal control without ratified treaties or reservation land, leaving their inherent rights to cultural burning unceeded, yet unrecognized and routinely disregarded (Clark et al. 2021; Thoreson 2023).

In the 1960s, the National Forest System's mission became one of multiple use, including timber production, watershed protection, recreation, and biodiversity. In practice, timber production dominated because of financial incentives (Hirt 1996; Wilkinson 2013; Biber 2008). On Karuk lands, Douglas fir *(Pseudotsuga menziesii)* plantations replaced hardwood forests, altering ecosystems and justifying fire suppression (Curtis 2022; K. M. Norgaard 2019). The Knudsen–Vandenberg Act, passed in 1930 and expanded in 1976, continues to incentivize timber sales by making a portion of the proceeds available to forest managers to spend on reforestation and improvement projects (Wilkinson 2013). These policies not only disrupted Karuk fire stewardship and lifeways by framing Karuk burning practices as an economic threat but also maintained the violence of settler colonialism against the Karuk people. This violence dates back to the early days of national forest management in the region. Orleans District Ranger F.W. Harley stated in 1918, about Karuk people: “The only way [to stop it] is to kill them off… every time you catch one sneaking around in the brush like a coyote, shoot at them” (as cited in K. M. Norgaard 2019, 98).

Early federal fire policies treated fire as destructive rather than a natural ecological process (Stephens and Ruth 2005; Pyne 2015). In Karuk territory, suppression tactics included water bombing and chemical drops, along with the use of Agent Orange derivatives 2,4‐D and 2,4,5‐T to suppress the growth of non‐conifer species; these chemicals contaminated food sources and caused reproductive health problems for Karuk women (K. M. Norgaard 2019). These harms compounded settler violence and displacement, which had already reduced the Karuk population to fewer than a thousand by the mid‐20th century. Federal occupation and suppression policies restricted access to ancestral lands and medicines, increased fuel loads, and undermined well‐being (Curtis 2022).

Decades of fire suppression and the absence of cultural burning left landscapes primed for high‐severity fires. The 2020 Slater Fire, which burned 157,000 acres of Karuk homelands, destroyed 200 homes and killed two firefighters, exemplifies the consequences of settler colonialism (Curtis 2022). Federal disregard for Karuk stewardship and criminalization of cultural burning altered fire regimes, facilitated invasive species spread, and increased fuel accumulation (Goode et al. 2022). The impacts on Karuk lifeways have been profound. Criminalization of cultural burning has disrupted basketry, ceremonies, traditional food cultivation, and the knowledge systems embedded in these practices. The loss of fire has reduced the prevalence of nutritious land‐based foods and medicines, increasing reliance on processed foods and associated health problems (Reid and Maestas 2019; K. M. Norgaard 2019). Invasive species outcompete native plants, Douglas fir monocultures reduce biodiversity, and denser forests increase smoke exposure and wildfire hazard (Marks‐Block et al. 2019; Anderson 2006). The Karuk relationship with their ancestral lands—once maintained through reciprocal stewardship—has been severely disrupted, creating an ecological imbalance that endangers both human and non‐human communities (Curtis 2022).

The impacts of colonization have forced the Karuk and other Indigenous communities to navigate federal funding structures to defend and strengthen land stewardship. Programs such as the Tribal Forest Protection Act (TFPA), the Stafford Act, and the Indian Self‐Determination and Education Assistance Act (ISDEAA) provide critical support for wildfire mitigation, ecosystem restoration, and self‐governance. Amendments in the 2018 Farm Bill and 2023 Bipartisan Infrastructure Law (BIL) expanded access to these funds, allowing greater autonomy in land management (U.S. Congress 2018; U.S. Department of the Interior 2021). Yet these funding structures are not without challenges. Federal funding timelines often conflict with traditional stewardship and seasonal responsibilities or include requirements that do not reflect community or ecological needs (Hoffman et al. 2022; U.S. Commission on Civil Rights 2003). Although these funding mechanisms offer critical support, the need for federal approval and partnerships can undermine Indigenous sovereignty.

### 1.3 Pathways for Restoring Indigenous Fire Stewardship

Through persistent advocacy, the Karuk Tribe has driven policy changes supporting cultural burning (Clark et al. 2021; Clark, Tripp, et al. 2024). For example, California Assembly Bill 642 legally defined cultural burning and created a state liaison, and Senate Bill 332 shifted liability standards from simple to gross negligence. Senate Bill 310 established sovereign‐to‐sovereign agreements recognizing Tribal authority over cultural burning and exempting Tribal Nations from state permits. However, as Nikolakis et al. (2025) note, laws by themselves are not enough to instigate social change; they require a variety of initiatives to reshape cultural molds, such as government support for cultural fire practitioners, eliminating agency silos, and advocating for Indigenous fire stewardship in management agencies and the public in general. For their part, the Karuk Tribe continues advocating for legislation that further upholds not only their rights but also the rights of other Tribes across the state, changing the culture of fire policy in California by creating more space for Indigenous expertise and leadership in forest and fire management. The Tribe is also working to shift the shared responsibility for wildfire by holding land managers accountable for the fire hazards created by fire suppression and the mismanagement of lands (Clark et al. 2021; Clark, Tripp, et al. 2024).

Karuk advocacy is part of a broader shift recognizing Indigenous fire stewardship as essential in the climate crisis (Tom et al. 2023; Vinyeta and Lynn 2013; Whyte 2017, 2018). Protecting Indigenous knowledge requires strong Indigenous data sovereignty measures accounting for related cultural practices, Indigenous rights, and ethical considerations (Hudson et al. 2023; David‐Chavez and Gavin 2018; David‐Chavez et al. 2024; Jennings et al. 2023). The Karuk Tribe has been at the forefront of this movement, restoring cultural burning while advancing Indigenous fire data sovereignty through research protocols, collaborative guidelines, and assertions of knowledge sovereignty (K. Norgaard 2014a, 2014b; Murveit et al. 2023; Thoreson 2023; Adams 2024). Unlike general ‘data sovereignty’—which often refers to state control over data within national borders—Indigenous data sovereignty emphasizes the rights of Indigenous peoples to govern how their knowledge, data, and cultural practices are collected, used, and shared. Karuk research efforts maintain cultural continuity, uphold Tribal values, and prevent extractive research practices (Sipnuuk Advisory Committee 2017). Research on intentional fire with the Karuk Tribe highlights its ecological benefits, including improved wildlife habitat, understory growth, reduced pest infestations, black oak and tanoak regeneration, willow restoration, increased acorn yields, and healthier soils (Connor et al. 2022; Marks‐Block et al. 2019; Halpern et al. 2022; Thoreson 2023; Lake 2007). It is our hope that this paper will add to the growing body of literature produced by the Karuk Tribe on the cultural and ecological necessities of Indigenous fire stewardship.

### 1.4 Summary

Pathways for restoring Indigenous fire stewardship are gaining momentum despite colonial legacies. Through advocacy and collaboration, the Karuk Tribe offers a model for restoring stewardship and asserting sovereignty without relying on agencies that historically excluded them from forest management (Coulthard 2014). Climate adaptation requires moving away from colonial land management toward Indigenous practices that recognize humans' integral role within the ecosystem. The absence of Indigenous fire stewardship threatens all living beings, human and non‐human, regardless of origin. Recognizing and protecting Tribal rights and knowledge is essential to reversing these trends. These understandings have informed the following research objectives for this project with the intent to develop a better understanding of Karuk perspectives, specifically regarding post‐fire recovery after the Slater Fire. Our research objectives were to: (1) identify post‐fire recovery priorities for the Karuk Tribe; (2) identify post‐fire management options to address Karuk priorities, and (3) characterize post‐fire governance systems affecting the Karuk Tribe.

## 2 Methods

We employed a case study approach to understand the unique experiences of the Karuk, a federally recognized Tribe, in recovering from a recent large (> 50,000 acre) and severe fire. This project was a collaboration between Colorado State University and the Karuk Department of Natural Resources (DNR). During the proposal stage, Principal Investigator Schultz consulted the Karuk Tribe's Research Coordinator to determine whether the project was of interest. Following the discussion, the Tribe issued a letter of support, expressing interest in the topic, its importance, and willingness to collaborate if funded. Once funded by the SW Climate Adaptation Science Center, a research committee was formed under a Practicing Pikyav agreement, as outlined in *Practicing Pikyav: Policy for Collaborative Projects and Research Initiatives with the Karuk Tribe* (Sipnuuk Advisory Committee 2017), which governs how the Tribe engages with outside researchers.

Initial efforts began in 2022, when principal investigator Schultz met with the Karuk advisory team: Heather Rickard and Shay Bourque (Karuk DNR), Kathy McCovey (Karuk elder, knowledge holder, and community liaison), and Bruno Seraphin (experienced outside researcher). The first field visits to Karuk territory occurred in 2023, when Schultz traveled to Orleans and Happy Camp, CA, to meet the advisory team, learn more about the landscape, and conduct initial interviews. This phase focused on relationship‐building. A second visit in 2024 featured lead author Cruz and Schultz conducting interviews in participants' homes (with permission) or at the Karuk DNR. Additional online interviews were conducted for those unavailable in person. Further visits occurred in July 2024 to present preliminary findings and in June 2025 to share results.

The Practicing Pikyav agreement guided all stages, ensuring cultural sensitivity and preventing extractive research. Consent documents allowed participants to choose whether interviews were recorded and confirmed their agreement to archive interviews with the Tribe and CSU. Participants were given the right to review their quotes, how they were used, and whether they wanted to be identified by name; all products were reviewed by Karuk DNR collaborators for accuracy. These steps ensure Tribal governance over data and accurate representation by outside researchers (Ramos 2021, 1). Cruz and Schultz coordinated with Shay Borque, Research Coordinator for the Karuk DNR's Pikyav Field Institute, to recruit participants and address questions before contacting Tribal members. In this role, Bourque, managed research requests and connected researchers with appropriate contacts, identifying individuals most suited to our questions. Participants also recommended others, whom we approached with permission. Participation was open to anyone with knowledge of fire response on federal lands relevant to the Karuk Tribe. We conducted 13 interviews with Karuk Tribal members and staff, recognizing that this small sample provides valuable insights but not the full range of experiences. Interviewees included:

- Will Harling, Mid Klamath Watershed Council Restoration Director (#1)
- Anonymous, Karuk Department of Natural Resources Employee (#2)
- Alex Watts‐Tobin, Karuk Tribal Historic Preservation Officer (#3)
- Earl Crosby, Retired Karuk Department of Natural Resources Employee (#4)
- Leaf Hillman, Karuk Ceremonial Leader (#5)
- Lisa Hillman, Karuk Tribal Member (#6)
- Michael Sanchez, Karuk Prescribed Fire and Fuels Specialist (#7)
- Erin Hillman, Karuk Tribe Director of Operations (#8)
- Vikki Preston, Karuk Tribe Cultural Resource Technician (#9)
- Anonymous, Karuk Tribal Employee (#10)
- Kathy McCovey, Karuk Cultural Practitioner (#11)
- Anonymous, Karuk Department of Natural Resources Employee (#12)
- Colleen Rossier, Karuk Tribe Senior Research and Policy Advisor (#13)

We conducted semi‐structured interviews, encouraging participants to guide the conversation and share experiences with fire, particularly the Slater Fire. Participants included Tribal members, retired Tribal employees, cultural practitioners, and fire and fisheries specialists. Our interview guide aligned with project goals, but participants were encouraged to raise issues they felt were most important. As a gesture of reciprocity, participants received pastries (recommended by Seraphin, who had prior experience working with the Tribe) and piñon pine nuts from Cruz's home community (Bouvier and MacDonald 2019). All non‐governmental employees were offered a $40 Amazon gift card, the maximum allowed by our institution, though not enough to compensate for people's time and knowledge; a donation was also made personally by Schultz to Karuk DNR in recognition of staff contributions, as staff could not accept gift cards.

With participants' consent, interviews were recorded, transcribed via Otter.ai, and reviewed for accuracy before archiving with CSU and the Tribe. Cruz and co‐author Russell developed a codebook using an emergent coding approach and reviewed several interviews to identify recurring themes, creating parent codes (e.g., “Governance”) and subcodes (e.g., “Collaboration,” “Consultation,” “Trust”). The finalized codebook, developed with input from all co‐authors, was refined through inter‐rater agreement (Campbell et al. 2013). Cruz coded all 13 interviews, exporting data to Excel and creating memos summarizing each code.

All quotations were approved by participants and Karuk Tribal leadership, with quotes identified by participant numbers or names, depending on interviewee's preferences. Member‐checking ensured participants could verify that findings respected and accurately reflected their knowledge. Research deliverables were tailored to Tribal priorities, including this peer‐reviewed publication, a 2024 Association for Fire Ecology conference presentation, a talk for the Tribe's Pikyáv Lecture Series and at the Klamath Fire Ecology Symposium in 2024, and a final presentation to the Karuk community in 2025. Although the study began with specific research questions, participants often focused on broader goals for fire management, using the Slater Fire as a starting point. Findings, therefore, are presented as thematic insights reflecting participants' priorities rather than direct answers to the original research questions.

## 3 Findings

This section is organized into two primary emergent themes that we identified during data analysis: (1) ongoing impacts of colonization in forest and fire management; and (2) revitalization of Karuk fire stewardship. These themes together are interrelated and tell a more holistic story, which we reflect upon in the discussion. For additional interview excerpts on the topics covered in the findings section, please refer to Table 1.

**TABLE 1:** Additional interview excerpts.

Theme	Quote
Site conversion and fire suppression	“Being four years out of that catastrophic fire, a lot more should have been done. I have a lot of concern for all these people who built homes back; they're at significant risk of them being burned down again. It's been infuriating” (#13).
	“The Forest Service had a unique opportunity, I thought, if they're going to make something out of this tragedy, they could have implemented something to where they put in place some sort of project to help put a buffer around the community, but I don't see it. I don't see the restoration” (#7).
	“They're able to suppress the good fires, then they're not able to suppress the bad ones” (#12).
	“Fire is a huge part of that catalyst for change and salmon tracking that change… If you think about fire suppression, you're not getting that catalyst for adding sediments, adding wood or all these spawning gravels to your channels. It's like, “we prevented this fire” but, you also prevented maybe all these good things from happening because of the fire” (#12).
	“The Forest Service has always been determined by commercial constraints. They work within what they think they can harvest and what they can sell; they are answerable to the mills and what the mills will accept, what the loggers can actually do. They answerable to that industry” (#3).
	“We [The Karuk Tribe] are adamantly opposed to the re‐establishment of conifer plantations” (#4). … “Since Forest Service has been around, if it burns, they're going to reestablish those plantations and not maintain them” (#4).
	“One of the leaders of the Orleans volunteer fire department first showed up on the scene as a Forest Service forester back in the 80s and her first timber sales were basically hack and squirt, poisoning these old growth groves of tan oak acorn gathering stands to convert them to conifers.” (#11).
	“Ours is a food forest and for so long it's been turned into a fiber forest, painfully for other people” (#11).
	“There are ecological processes at play that have adapted to fire being part of the landscape. The management paradigm and the dominion over nature mentality, just pushes back against that and causes more problems than does good in a lot of ways” (#2).
	“It's this perverse incentive that is a legacy of a timber economy that incentivizes the wrong stuff at the wrong time. Those days are so far behind, last thing we need to be doing is incentivizing the destruction of our forests, which is exactly what it comes down to. I say that in advance, talking about the Slater Fire, because the Slater Fire didn't have to happen but, it was inevitable, unless some action was taken to prevent it. It was predictable” (#5).
	“Mismanagement of a forest that is making fire hazard for communities for human life. They're making it worse, not better. They're doing nothing to mitigate those increased hazards for communities. No prescribed fire at all” (#5).
Differences between Federal Agency and Indigenous Worldviews	“When you live in a place for 10,000 years, you figure some things out. You learn how to manage it, not how much damage you can do and for it to barely survive. Which is how we do our Environmental Impact Statements–how bad can we treat this land and have it not just fall apart completely? Manage for the minimum thresholds. When we manage for abundance instead, which is how we have always done, we manage everything for abundance it's not a lowest common denominator” (#5).
	“Western science hasn't been able to grasp the processes that are occurring in this place, the fires, the floods, the windstorms, the thunderstorms, the change agents that are affecting and shaping these habitats. They failed to account for humans” (#2).
Collaboration	“Resources, at least with the Forest Service, are really limited these days. When they bring in big teams, they'll bring in a fish biologist from some other part of the country that has no personal connection or no connection or doesn't really appreciate the types of streams we have here within the Klamath. One of my suggestions would be is having more interaction with the local Tribal biologists, fish biologists, wildlife, those kinds of folks” (#12).
	“You can't trust them [Forest Service] not to do something that they're not supposed to do” (#4).
	“Post‐fire, it's like you're resistant to identify cultural items [to federal employees] because you don't know how that's going to be used” (#4).
	“There was no planning of any diverse species to get some of those rare and endemic conifers that the Indian Creek watershed is known for. They [Forest Service] just left it, they abandoned it. Just like, “We can't do anything except for log it.” ” (#4).
	“I think that the Forest Service should be embracing a wholesale shift in fire policy that mirrors Indigenous fire management in the Klamath mountains, because there's no other workable solution than frequent fire in these post fire landscapes, and every fire scar is an opportunity to get it right. And yet, we continue to see the agencies missing those opportunities by not conducting the appropriate NEPA or not preparing their constituent groups for that reality. And we're about to miss the boat on the Slater fire, which would be an incredible tragedy” (#1).
	“The Six Rivers has embraced collaboration on a much deeper sense than the Klamath because it's a mechanism for them to receive more funding. [Six Rivers] is not as beholden to timber resources” (#1).
	“That Klamath has always retained the ability no matter what the collaborative says, to make their own decision and do whatever they want, at the end of the day, whereas the Six Rivers and their line officers have repeatedly shown through their words and actions, that they're only going to do projects that the [collaborators] fully support. And that is a huge and transformative act right there” (#1).
	“The approach that we're doing on Six Rivers [National Forest] systemically puts fires back, in the approach from the Klamath National Forests appears to be more about making it cheaper to put fires out, safer to put fires out, and so it's two completely opposite approaches. It's a shame that we can't get through our issues, for our Tribal people that live in our territory and in our community because of that” (#2).
	“The Klamath National Forest just does not seem to like to allow the Tribe to have any kind of meaningful involvement. Whereas on the Six Rivers… were going to come to an agreement on how we're going to do this together. One unit is one lane, and one unit just kind of the opposite, which is frustrating, to say the least” (#2).
	“The Six Rivers [National Forest], they're definitely more strategic than on the Klamath. I was on the Klamath and they were really trying to put the fire out as soon as they could. Here [on the Six Rivers] they use a different tactic; they won't defer the risk” (#7).
Funding	“Incentives within the agency are stacked against the use of managed wildfire…there's a lot of liability and not a lot of gain” (#2).
	“The Klamath has at least three NEPA teams… the funding for those teams is based on their timber outputs, [they're] the major producer of timber in Region Five of the US Forest Service, whereas the Six Rivers National Forest produces less timber largely because they live in a more liberal area with a lot of the communities like Arcata, Humboldt, Cal Poly and Humboldt. They're not able to get out to cut… they have barely one NEPA team. So the funding that the forest gets, historically, was based on getting out to cut” (#1).
	“The BIA is supposed to be helping Tribes govern these things themselves, but if Congress doesn't allocate the money to be able to enable programs to be built, then it just doesn't happen” (#2).
Karuk Leadership, Knowledge, Experience	“The Native people survived here by gathering food that they could store… This is this is our garden of Eden… To survive, we have to use some type of tool that's cost‐effective; it's also a built‐in mechanism that these trees have evolved to… Fire is our tool to inhibit plants. The use of fire, when we use it, how we use it, where we use it, etc., that will help dictate what comes back” (#11).
	“You try to find the largest trees that are surviving, you get different cohorts, you make sure they're well‐spaced and the brush doesn't come back in, so, you have the shaded fuel break where the fire comes up. It can get back on the ground again, it has the ability to slow down a fire, certain conditions, and certain fire events” (#4).
	“It's a multiple entry, it's not burn it once. Burn it the first time… you wait a couple years to get enough fuel to burn again until you can reestablish a mosaic of different species that come back after fire and try to coppice some of the oaks that come back to the harvests, multiple stems. We're trying to coppice those and pick two or three of the best stems and get those to create, a new tree comes from the sub sprouts” (#4).
	“It's never too late to burn. You could burn at the right time, right place, it just takes that political way to do it” (#4).
	“We don't just care about tangible things, like an artifact or a prayer spot… it's a whole landscape that the Tribes cares about, is concerned about, and has a duty since time immemorial to manage it and protect it” (#4).
	“I don't care how many 747's they turn into fire tankers, what your capacity is and how many remote sensing devices are out there. We're not going to buy our way out of this with technology or more fire apparatus. It's not possible to win a war if you're fighting fire. It's not possible to win. The whole concept has to be changed. People have to view fire differently. Not as a fight. Not as a war. You'll lose every time. You can't fight fire. You have to work with fire. Fire has to be your friend. People have to have a relationship with the fire” (#5).
	“The land is asking for fire back in a good way and we have to do that by any means necessary” (#2).
	“These open areas, if they don't get the fire, they don't produce. That's a food forest. My grandpa, we always used to go, my grandma, into clear cuts because the hazel would come back and that's where he would find his hazelnuts” (#11).
	“The Karuk Good Fire policy report has been an amazing resource for having direct input into the state's prescribed fire and managed wildfire plan (#5).”
	“This community is different. The people that live here are different. It's part of the culture. Fire is part of it. People have a relationship with fire. Turns out this is a rare thing that doesn't happen an hour drive down the road. Its like a different world” (#5).
	“We need to reach to reconnect our knowledge, practice, and belief systems to that landscape. And not just that landscape, but other landscapes we don't want to see that happen too” (#2).
	“When you have a hard freeze, those windows are great burning windows because fire will carry during a freeze in the middle of winter. You can actually get some acres done midwinter if you're following the weather and you're paying attention, you can get those things done. Recognize that we're not going to get those things done in the middle of summer. It's not going to happen, it's too dangerous to do but, we can start nibbling away at it” (#5).
	“[Ceremonial] fires burned until the rains put them out. They could because they just creeped around; they didn't do anything. Part of that was managing the fish runs, because it caused an inversion to send into these valleys. The inversion drops the temperature of the river and triggers the fish to run. Not rocket science but Indigenous science for sure” (#5).
	“People have families, nobody wants to spend the rest of their life in prison trying to do what is their God given right to do, or even their responsibility to do, even their religion to do. Nobody wants to spend the rest of your life away from your kids and your family, right? Decriminalizing these actions [of using fire] and providing support for people to do the right thing. Now, those kinds of things are supporting people, recognizing that people aren't trying to be criminals. They shouldn't be treated like criminals, they should be treated as human beings, supported in their actions because their actions are the ones that's gonna save everyone else” (#5).
	“Karuk can be a leader, not just for Karuk territory but to share experience and lessons learned with a lot of other Tribes too. At KDNR, we have a lot of experience dealing with the federal government and Forest Service. It can be quite challenging” (#13).
	“Wilderness had been shaped by human hand, by Karuk hands for 1000s of years” (#5).
	“Just like cultural burns, intentional, our presence here is intentional, everything we do” (#5).

### 3.1 Ongoing Impacts of Colonization in Forest and Fire Management

Throughout multiple interviews, participants referred to the impacts of settler colonialism on Karuk ancestral territory that have and continue to disrupt Karuk fire stewardship practices. One participant recalled an early Forest Service policy requiring Karuk people to apply for immunity allotments. This policy was intended for the Karuk Peoples to avoid accusations of trespassing on their ancestral territories on what had become National Forest land. Leaf Hillman, a Karuk Ceremonial Leader, explained:

The people didn't want land allotted to them because they didn't need someone to say, ‘You can own this land.’ The [Karuk] never owned the land. They had a responsibility to manage a much larger place. The whole hillsides behind their entire mountainside were their responsibility to manage…. From that day forward, these forests have suffered from the lack of human touch. It's been purposely rubbed out (#5).

They described the current ecological state of their ancestral territory as unhealthy, severely out of balance, and highly vulnerable to wildfire. They emphasized that the Karuk people have always sought to care for the land, but their stewardship practices have been systematically suppressed and threatened by the current legal system. Hillman further expressed, “I know how to do [cultural burning], but…I need to count on the fact that the sheriff isn't going to show up and try to arrest me, or the Forest Service isn't going to roll on it and suppress it” (#5). Hillman continued, “If you don't intentionally set out to [burn] and you let the damn thing grow back however it wants to without human intervention, what you're going to get is a conifer dominated forest. That isn't the nature of these forests around here. It hasn't been for 10,000 years…. There were different parties that were responsible for managing those resources. The doctor people, the healers, they managed the medicinal plants. Basket weavers managed the hazels and willows, all those kinds of things. The men managed the hunting grounds in the high country. Men and women managed the acorn groves in the middle elevations. We didn't have to protect our community from escaped wildfire because there was no escaped wildfire. There was no buildup of fuels that was unattended because it was somebody's business to manage those fuels” (#5). All participants echoed this, often describing humans and fire as a keystone species and essential elements for ecosystems, noting how this contrasts with Western worldviews.

Many current and former Karuk DNR employees discussed the broader federal management system that prioritizes short‐term economic and developmental gains through the timber industry. According to participants and other historical accounts (see K. M. Norgaard 2019), the Forest Service suppressed and killed native oak species while planting and facilitating the growth of Douglas fir (Pseudotsuga menziesii) for timber production. They said that Douglas fir, without fire, outcompetes native vegetation like black oaks *(Quercus velutina)*, which are crucial for the Karuk community's diet and the ecosystem's balance. Kathy McCovey, a Karuk cultural practitioner and retired Forest Service employee, explained that these practices stemmed from congressional mandates, explaining, “The Forest Service is always trying to kill oaks…. These trees are here for a reason, they want to grow here… I was a silviculturist for the Forest Service. My job was to go out and do timber sales. I would get a letter from Congress via the Forest Service, ‘You are to cut between 2 million and 4 million board feet of timber. You are to regenerate 200 acres of land into Douglas fir trees for the future” (#11).

Participants specifically attributed the Slater Fire's severity to Forest Service land management practices. Leaf Hillman stated: “Slater Fire, lay that one 100% at the feet of the Forest Service and their fire suppression policy and timber management policy…. They cut every stick of timber for miles around, creating even‐aged forests, densely packed. They've suppressed every fire, every fire that popped up in that zone has been attacked and snuffed out” (#5). Another DNR employee said: “This is the type of event we're going to be seeing more of in the future. A lot of people say, ‘Oh, that's climate change.’ [But] we've always had northeast wind events. [What] we haven't had is 100 years of fuel loading, and we haven't had a sea of plantations on the northeast side of town that align with those wind events. That just makes fires huge, throw embers two miles and burn completely, all at once” (#2).

Despite reoccurring wildfires in the region, participants explained that the Forest Service continues to practice timber extraction and site conversion during post‐fire restoration efforts, perpetuating a cycle of wildfire vulnerability. An employee at Karuk DNR explained, “Looking at this landscape, you can see where small fires were, you can see where big fires were, you can see hardwood stands in those old footprints and you can see them surrounded by sea of conifers. You can just imagine how long it will be before that burns, takes out this whole side of town with it; it is just a matter of time” (#2). They explained that within seven to 10 years, they expect other competing vegetation to be removed to maintain the Douglas fir plantations for future harvests, typically scheduled every 75 years. Yet, according to participants, major fires have occurred more frequently in the area, often burning trees prior to them reaching maturity for harvest (Odion et al. 2004; Shatford et al. 2007). Will Harling shared, “Seventy percent of the plantations in the Salmon River watershed, which is 751 square miles, have burned before they reached merchantable age, and yet they continue those practices. Today we hear the Forest Service saying they're interested in putting fire back into recent fire footprints; however, we have yet to see it happen” (#1). McCovey observed that this is in line with the training foresters receive and also that many federal employees leave the area before they learn about the broader ecology and fire behavior, never truly realizing the impacts of their practices. Karuk DNR employees noted that National Forests with higher timber yields typically receive increased budgets and staffing from the Washington office, whereas critical fire management practices, such as prescribed burning, are deprioritized. Another Karuk DNR employee explained: “We should be looking at what's trying to come back on its own before we just start thinking we know better than nature…. That first seven years [after fire] should be about ‘Okay, where do we need to get fire back in?’ If you go planting trees [then, the forest] becomes an investment to protect. The next time there's a fire; it becomes a justification to put the fire out. … putting fire back in? That is going to be the only solution” (#2).

Lisa Hillman, a Karuk Tribal member, also expressed frustration that fire mitigation and burning efforts that would benefit the land and improve community safety are only addressed if there is leftover funding after timber production and fire suppression efforts: “The [Forest Service staff] go and they harvest, and slash. [Then they say] they'll come back later if there's resources, if there's time and money for it but no. It sets us up for another catastrophic fire” (#6). She continued, “Now we're a tinderbox. We can't get fire insurance. Those who did have fire insurance were dropped right before the Slater Fire. So, what happens is I'm homeless, but it's not my fault” (#6).

Most participants explained cultural burning considers other holistic ecosystem factors such as biodiversity, watershed stability, and the health of culturally significant species, whereas standard fire suppression and federal policies like the Endangered Species Act (ESA) focus on a single species or variable at a time. Will Harling explained, “Western science looks at the world through the lens of an individual species at a time; it misses the lessons of deep time, generations of animals, plants, or the type of knowledge that you only get by living in a place for millennia” (#1).

Reflecting upon the Slater Fire and other fires, many participants described federal land management as an ongoing settler colonial project of cultural oppression that disconnects Karuk people from their lands. They explained that fire suppression tactics, coupled with increased drought and extreme fire conditions exacerbated by climate change, have reduced the quality and abundance of vital cultural resources used for ceremonies and everyday needs, such as basket materials, foods, and medicine, making it harder to maintain cultural traditions. Leaf Hillman explained: “That's one of the hardest things about it…that fire burns so hot you are under threat of losing connection to those plants. When the materials are no good that disincentivizes people from going up there and tending and harvesting.” (#5). Interviewees also said that high‐severity wildfires have forced Karuk families to leave their ancestral lands, often relocating to more affordable areas where they can rebuild or gather essential resources, a situation they view as a continuation of historical displacement. Erin Hillman, Director of Operations for the Karuk Tribe and Tribal member who lost her home in the Slater Fire stated, “It's like being chased out of your homeland. It's like, nobody respects how important that is, a people that is from this area, wants to stay in this area…. But people have had to leave because they can't stay. So, what does that do to our Tribal culture?” (#8).

Many participants spoke on how Karuk people must continually seek external approval to carry out their cultural heritage. They explained that fire is element of their religion, one that is neither recognized nor protected because it involves fire and land management, despite freedom of religion provisions in the U.S. Constitution. Hillman explained, “Our religion isn't protected under the Constitution. Our religion isn't protected because fire is part of our religion” (#5). Some explained that the Karuk Tribe intentionally eschews requirements to seek permission from federal agencies like the Forest Service when it is an unceded right on unceded territory and fundamental to their religious freedom. Although federal agencies have made some strides toward using intentional fire for fuels reduction, people said that seasonal limitations often result in missed burn windows. One participant explained, “According to the law, it is flat out illegal to burn in our traditional time of year. The only thing that will burn in [Forest Service burning seasons] are those extremely dry locations. The whole system is designed to work against what we need to do” (#2). Michael Sanchez, a Prescribed Fire and Fuels Specialist for Karuk DNR, said, “The lack of allowing fires to burn during suitable windows has created an intense footprint once the fires pass through. It's progressively gotten worse; every year brings a new ‘biggest fire.’ In the summers, there's miles without a green tree” (#7). They went on to explain other bureaucratic barriers, such as the Limited Operating Periods (LOPs) for Northern Spotted Owl ( *Strix occidentalis* ) conservation. They stated: “One of the biggest issues [the Tribe] has is the LOPs. That winter burn window cuts [burning] off at February 1 to the end of March, where we could do a lot of positive burning. The spotted owl, for the Tribe, isn't a focal species. We don't have a Karuk name and vocabulary for the spotted owl; it doesn't have huge importance to us…. We've been really trying to push towards cultural burning, trying to get past the policies and the hiccups of being able to go out and do burning with a lot less resources than their law requires” (#7). Individuals noted too that they did not believe their burning efforts would be detrimental to the species overall and would benefit the ecosystem more broadly.

Many interviewees expressed that post‐fire is an opportunity for federal land management systems to recognize Karuk rights and knowledge systems and to work with Karuk to reintroduce fire to their ancestral lands. However, following the Slater Fire, they noticed that private landowners have taken action to manage the land, but that Forest Service land remains largely untouched and overgrown, except for some instances of salvage logging. Karuk DNR employees said they have observed that rather than burning or establishing fuel breaks, the Forest Service exploits the emergency status of wildfires to bypass environmental compliance, like under the National Environmental Policy Act (NEPA), fast‐tracking timber extraction projects under emergency declarations. One person explained: “The 2020 Slater fire and the 2022 McKinney fire both had extensive salvage logging efforts afterwards. Heavy equipment on severely impacted and sensitive soils from high severity fires is never a good combination…. Post‐Slater fire, they logged a 600‐foot‐wide swath along the Gray Back Road that goes from Happy Camp over into Oregon, 600‐feet wide by, 30 miles long. It's a lot of trees, way more than they get out during their planned timber sales and without any environmental compliance. They're utilizing that emergency loophole created by wildfires to get out the cut in a large way on Klamath National Forest” (#12).

They explained how the Forest Service can also use post‐fire management funds to support timber sales. Earl Crosby, a retired DNR employee, stated: “They have people out there on the ground laying these timber sales out before the fire is out. You want to talk about their recovery and all that stuff, it's all complete bullshit. All they're doing with those funds, suppression repair costs and the BAER funds, are supporting timber sales every year for the last four years.” (#4). A Karuk DNR employee also highlighted that federal post‐fire practices meant to prioritize hazard tree removal for road clearing and salvage logging can often disregard the ecological balance of sensitive stream channels. They explained that tree removal destabilizes soils that support water channels utilized by the Karuk and local species, leading to erosion, muddy waters and damaging freshwater ecosystems. The expert explained, “You don't necessarily need to remove these trees, you can directionally fell them into the channels and still mitigate the hazard. These stream channels need to recover, and we're setting them back by having post‐fire suppression repair that doesn't consider the health of the stream channels” (#12).

In summary, one Karuk member explained, “Every time they exclude a fire, they're causing more fuel to build up, and so everybody ties liability to the ignition. That gives agencies a free pass. Where [by contrast] the cause of the impact can be tied more directly to the fact that fire has been excluded, more than the fact that a fire has occurred” (#2).

### 3.2 Revitalizing Karuk Fire Stewardship

Today, the Karuk Tribe is working to assert its sovereign right to burn without federal oversight. As Leaf Hillman put it, “We can't sit by and allow it to be mismanaged the way that it has been. We have to speak out loud, say it often, and to anyone who will listen” (#5). One elder stated, “We're very lucky that we have retained so much of that traditional knowledge about our ceremonies. We've brought those back. The pieces that we haven't brought back are the fire pieces that are critical components to those annual ceremonies” (#5). Many participants linked eco‐cultural revitalization to the need for federal agencies to recognize Karuk sovereignty and their inherent right to burn. They stressed that for both their land and community to heal after high‐severity wildfires, Karuk‐led burning practices must be restored without criminalization or external constraints. Will Harling explained, “every wildfire is an opportunity to restore cultural fire and to prepare for the next wildfire on that landscape in a way that is accepting the frequent fire instead of excluding it” (#1).

Efforts to restore cultural fire practices and strengthen connections to ancestral lands include forest management practices, such as coppicing (repeatedly cutting back tree trunks to ground level). Kathy McCovey explained that coppicing promotes the growth of new and stronger stems for native hardwoods, potentially making them more resilient to wildfire. McCovey explained that these efforts not only prepare and strengthen the landscape for wildfire but also repair their connection to the land. They explained, “This is going to become an area where we bring our kids. Our kids are scared of fire now because half the town burned up, but, if we come in and we start [coppicing], we have [cultural] burns that prepare for fires coming towards us, if we prepare our areas to receive fire it may not be such a traumatic event” (#11).

Employees of the Karuk DNR explained that their ancestral lands span two National Forests, Klamath and Six Rivers, requiring them to work with federal agencies to implement land management projects. They have initiated plans for small‐scale burns within the Slater Fire footprint as part of their broader strategy to revive fire stewardship. These efforts are hindered by the lengthy NEPA process required to burn, which members explained often delays or prevents the Tribe from meeting specific burn windows. In addition to NEPA, cultural practitioners must complete detailed burn plans. These federal requirements are barriers to fire stewardship work, said interviewees.

Participants explained that the Tribe's collaboration efforts proceed differently in the two forests, complicating the Tribe's ability to manage the land effectively. The Six Rivers National Forest was described as more supportive of Tribal fire stewardship, with more engagement from leadership and their staff. In contrast, participants described collaboration experiences with the Klamath National Forest as “box‐checking” exercises by the agency, whereby the National Forest notifies the Tribe of predetermined plans without meaningful dialog or consideration of Tribal input. Will Harling explained the reality of federal acknowledgement and “inclusion” of Indigenous Knowledges in land management, saying, “They'll praise [collaboration] in their public presentations, but when actually implementing it into their practices, it just doesn't seem to exist” (#1). Participants explained that a lack of engagement hinders the co‐development of any innovative land management project on ancestral lands. Earl Crosby explained that by the time the Karuk Tribe is engaged, “The [Forest Service] is so far down the pipe on the planning that they are not willing to reverse gears and think about a different approach” (#4). Participants consistently attributed this perspective to a reflection of individual leadership, county politics, and land management history. Additionally, participants highlighted challenges with outsiders who join the Forest Service primarily for career advancement, rather than with a genuine commitment to supporting and collaborating with the Karuk people. Kathy McCovey observed, “People don't come because they want to be here; they come here for [advancement] in their job. They're disassociated with the community” (#11).

Participants noted that restoring ancestral knowledge and cultural burning on their ancestral lands is also hindered by individual understanding and attitudes of federal employees. As a result, Forest Service personnel with a Tribal background or history of working with the Tribe often are more collaborative. DNR employees explained that during fire‐related incidents on Karuk ancestral lands, authority is typically granted to the Forest Service or state fire departments like the California Department of Forestry and Fire Protection (CAL FIRE). They noted that these agencies often deploy personnel or fire crews unfamiliar with the local area, its unique ecological conditions, or even the existence of the Tribe. Participants attributed the lack of awareness to the absence and acknowledgement of Karuk history and land stewardship in education and training programs. Many participants explained that these dynamics shape the attitudes and approaches of decision‐makers and collaborators, further excluding Karuk knowledge systems and practices in federal fire management. Several participants noted encountering resistance or a lack of willingness to collaborate from agency decision‐makers. Another participant noted that the Tribe was able to influence the incident management team on a fire, but it required persistent repetition. They said, “After a while, we did it, but you get tired. Every 14 days, a new team would come in. You'd have to do the same thing all over again and go through your spiel, fight the same fights, all over again” (#4).

Most participants attributed challenges to people's lack of understanding and experience with the Tribe, but a few interviewees mentioned some specific examples of explicit racism. One example occurred on a fire line in Happy Camp, where a bulldozer damaged a cultural site. When asked why they think that happened, the participant stated that it was intentional on the basis of the identification of the cultural sites during fire management briefings. The participant explained, “The older cadre firmly believed that it was the [federal government's] forest. We, as the Tribe, had no say in how they managed their forest, they figured it was their land. The old timers [say], ‘This is our land. This is federal government land and we're going to do what we think is best. You guys are just another party, interested public.’ They couldn't wrap it around their head that the Tribe was interested in more than what was on the reservation, they are interested in the whole landscape within the Aboriginal territory” (#4).

Other participants shared observations of sexism during wildfire incidents, explaining that outside collaborators typically speak and engage only with male counterparts. Some noted that gender identity is a specific target of settler colonialism, where the authority and knowledge of Indigenous women and Two‐Spirit people are systematically undermined to reinforce settler control over land, leadership, and knowledge systems. Leaf Hillman recalled numerous encounters with an incident management team where the Tribal Representative, a woman, was ignored by agency personnel. They continued to explain that misogyny in wildfire incidents is not just at the local level but is an agency issue. They stated: “It's not a place for women to be. Women get harassed and get driven out of those kind of places and spaces. It's not a friendly place. We need more people thinking, not just making decisions based on their testosterone levels… The whole apparatus is a male‐dominated, adrenaline‐fueled mess. We need more women to be in decision making spaces on the forests and in these incident management teams” (#5).

Another participant emphasized that lawsuits against the Klamath National Forest are often the Tribe's only means of having their input considered in land management decisions. They referenced the Memorandum of Understanding (MOU) between the Karuk Tribe and both the Six Rivers and Klamath National Forests, which outlines a shared commitment to “jointly identify, plan, and accomplish mutually beneficial projects, such as watershed restoration, job creation, and community economic development” (Karuk Tribe and U.S. Forest Service 2000). The participants also discussed the interdisciplinary team responsible for developing the forest plan for these National Forests. Reflecting on their years of experience in Karuk DNR, Leaf Hillman explained: “The successes we've had all started during times when they [the Forest Service] were under a lawsuit. That's how we ended up with a fire MOU in the first place, on the tail end of a lawsuit. That's how we got someone on the ID [interdisciplinary] team for the forest plan. Anytime we made progress in our relationship, it was because of a lawsuit. We had their attention for a while, but it wears off, and they have to be reminded pretty frequently” (#5). Yet the significance of the Karuk Tribe as the original stewards of the land is also disregarded through frequent turnover in agency leadership, often leaving Karuk stewardship projects at the mercy of external bureaucratic powers beyond their control. As Colleen Rossier noted, “The risk is if someone changes, who's in a leadership role there, that throws our whole program into some risk” (#13).

Although collaboration with non‐Tribal entities often presents challenges, participants shared a positive outcome that occurred during the 2023 Six Rivers Lightning Complex. Through the Integrated Strategic Action Plan (ISAP) process, the Karuk Tribe worked with federal agency leaders to transition from fire suppression tactics to strategic fire use during the incident. Leaf Hillman explained that these fires were caused by lightning and were burning far from communities, and with resources already stretched thin because of other wildfires, the Tribe and leaders in the Forest Service who were deployed to manage the fire saw an opportunity to utilize the natural ignitions to reduce fuels in those areas. He said, “It's really an anomaly that happened, right people…right time, right place. It wasn't easy to take advantage of that opportunity…. We had people on the incident who were, these hotshot crews particularly, just shake their heads, like, ‘What are we doing? Why are we not suppressing this fire? We have an opportunity. We can go direct and we can put this thing out.’ It's like, you're missing the point. That's not what we want to do. We're getting good burning; we're getting good fire on the ground. Why would we defer that risk? It's a lack of understanding and they've been educated to do, and trained, and drummed into them, that this is the way you do it” (#5). The Karuk DNR documented these efforts during the Lightning Complex in a short video, intentionally wanting to use it as a teaching tool to advocate for cultural fire practices in the future (Karuk Media 2024). Hillman reflected, “We need to take the opportunity, take advantage of something good that happened, and document it. Then use it to teach these teams” (#5).

Participants also highlighted the critical role of funding in revitalizing Karuk fire stewardship, noting the Tribe faces significant challenges in securing and implementing adequate funding needed for resources used in land management projects. DNR employees explained that the Tribe relies heavily on grants because existing funding from the federal government, often calculated on the basis of acreage of trust land or tied to specific constraints and deliverables, is insufficient to meet their land management and staffing needs. Will Harling described the limitations of this funding, stating: “[The Karuk Tribe] gets about $1200 a year for community wildfire coordination. It's National Fire Plan money, based on some formula they established using acres. So, we get $1200 a year as our base funding for our fire program. You can't even pay the heating bill with that, basically. We're forced to depend on grants because all of these federal programs have allocations and budgets tied to them. We try to align our staffing at commensurate levels, but our staff has to do a lot more work than the federal staff, because we have to write grants, we have to do all these other things. And so, there's a significant equity issue there” (#1).

Participants explained that, therefore, Karuk DNR relies heavily on a combination of state, federal, and private grants to sustain their fire programs and staff to return fire to the landscape. A few participants mentioned that Tribal Forest Protection Act of 2004 (TFPA) funding is particularly helpful in returning fire, as it supports both planning and implementation efforts and provides liability coverage for burn bosses under the 638‐contracting authority, resulting in Tribal workers being treated as federal employees. The 638‐contract, established under the Indian Self‐Determination and Education Assistance Act of 1975 (ISDEAA), enables Tribal Nations to have authority over federal programs and services, like fire management, enhancing overall self‐governance and workforce protections. In contrast, they explained that the Collaborative Forest Landscape Restoration Program (CFLRP) funding they receive is more implementation‐focused and less flexible in terms of who can access or utilize the funding. CFLRP is a federal initiative intended to help restoration efforts across large, fire‐adapted landscapes through collaborative projects with various stakeholders, federal agencies, and rights‐holders, such as Tribal Nations. One participant explained,

We're building in [TFPA] funding for our burn bosses that are going to be implementing prescribed fire and for our folks that are going to be overseeing or coordinating with cultural practitioners to help govern the actions of our membership or our citizen base. This is so cultural fire practitioners can do what they need to do outside of those systems with NWCG qualifications and permits and all that. Hopefully we can work directly with the feds and the states on the coordination of that and make sure that we've got proper notification procedures going on, everyone knows what's happening, and we're not just having a bunch of crazy things happening that scare everybody, but ultimately, we have to live with some level of cautiousness as well (#2).

Participants also recounted a recent event where Karuk DNR received $11.5 million through the Wildfire Crisis Strategy (WCS), which was begun by the US Forest Service after passage of the Bipartisan Infrastructure Law in 2021 (Forest Service 2022). The Klamath River Basin was designated as a priority area for investment in 2022. Participants explained that $1.5 million was allocated for Forest‐wide NEPA planning related to fire, fuels, and aquatic systems, whereas $4 million was designated for hazardous fuels reduction projects, such as cut‐and‐pile treatments and future broadcast burning in the Klamath National Forest. We note, however, that all WCS funds were put on hold during federal fiscal year 2025 and that multiple sources of federal funding are currently in flux in response to the Trump administration's executive orders.

Participants stated that Karuk DNR also benefits from programs like the Bureau of Indian Affairs' (BIA) Reserved Treaty Rights Lands (RTRL) program, which provides funding to federally recognized Tribal Nations for collaborations with federal, state, private, and other landowners for land management projects aimed at treating areas of ancestral territory with high wildfire risk (Russell et al. 2021). However, they explained that BIA funding intended for fire‐related projects is usually minimal and is calculated on the basis of the acreage of trust land. The Karuk Tribe only has 900 acres of their ancestral territory in trust and this land had to be acquired first and then worked through the fee‐to‐trust land program. The fee‐to‐trust land program helps federally recognized Tribal Nations place “fee simple” lands into trust lands intended to benefit the Tribe with protections and programs under the BIA (Quinn 2015). They mentioned that BIA funds are helpful for training, burn plans, and overtime costs for Tribal workers. However, BIA's involvement in daily operations is minimal because of low capacity, which slows down the burn approval process, according to participants.

Despite significant challenges with funding and collaboration, participants highlighted the Karuk Tribe's ongoing efforts to restore cultural fire practices and strengthen their connection to ancestral lands. A product of their efforts includes *Good Fire I and II* (Clark et al. 2021; Clark, Tripp, et al. 2024). These documents cover bureaucratic barriers, management options, and ecological benefits to cultural burning in California, advocating for policy reform at the state and federal levels. The documents also emphasize a difference between prescribed fire and cultural burning, a topic that was mentioned throughout multiple interviews. Although the primary difference between prescribed fire and cultural burning can be seen as applying fire to the landscape for fuel reduction versus its holistic benefits, participants pointed out that cultural burning does not include government resources, approvals, or permits, whereas prescribed fire does. One participant stated, “Cultural burning is done within the sovereign authority of the Karuk people, or the sovereign responsibility of the Karuk people. Prescribed fire fits within the context of those other governance systems” (#2). Michael Sanchez further stated:

People have different definitions of cultural burning. Each Tribe has their own definition. Ours is strict, we're not going to ask the Forest Service for anything. We're not going to do a permit or anything like that. We don't need to ask. We don't need to fill out this form to do cultural burning. We're at a standstill about progressing…. where we need to be is in black oak stands in February and March, and be able to burn it with one person, not spend all the money on putting a line around it and having engines all over, people all over. It's not cost effective. You can go up to a black oak stand surrounded by fir trees, light it, and it'll go out once it goes into the fir trees (#7).

**FIGURE 1:**
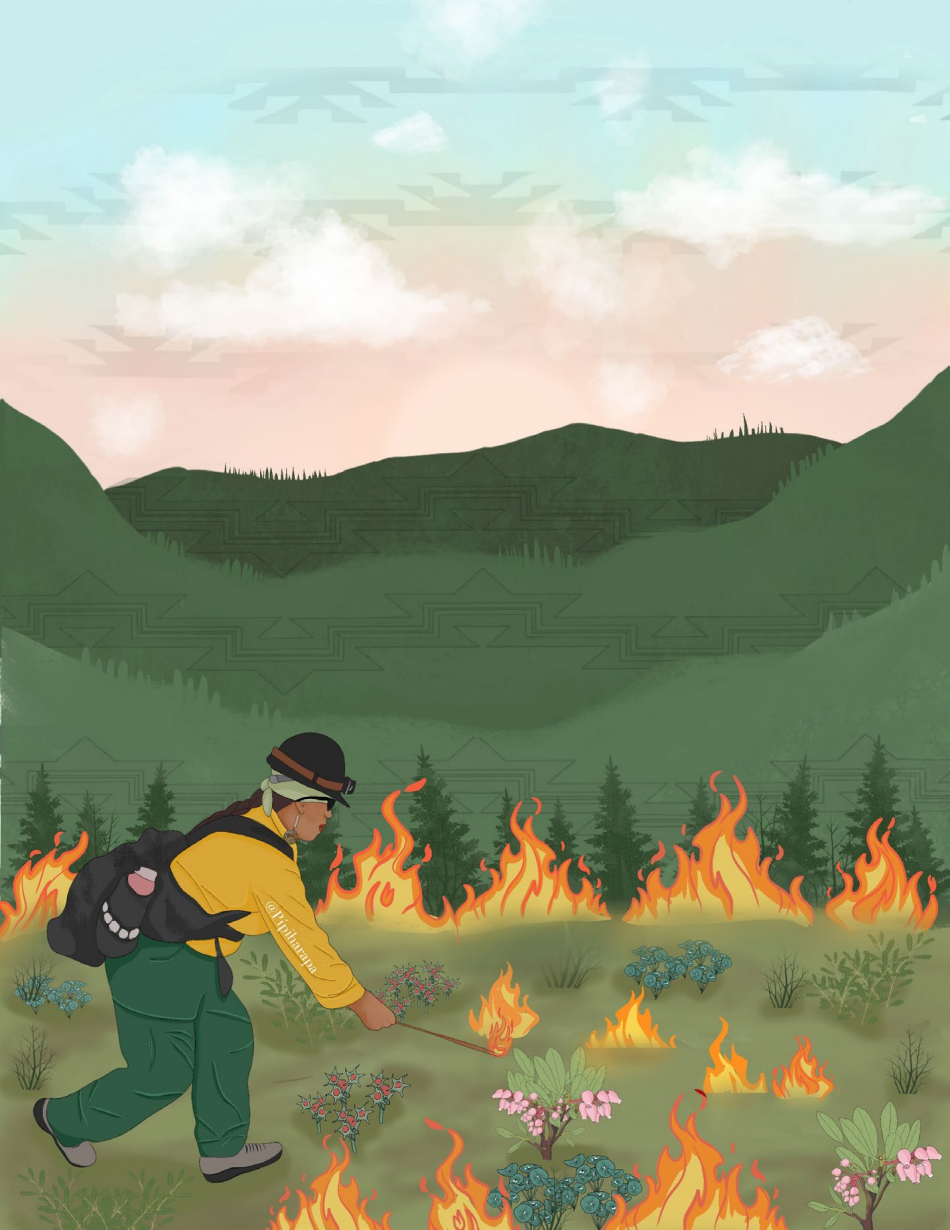
Karuk Fire Practitioner by Xatímniim Drake.

In addition to *Good Fire I and II*, participants noted that the Karuk Tribe works to provide opportunities to learn and practice cultural burning through the Karuk Indigenous Women‐in‐Fire Training Exchange (TREX). The Karuk TREX began in 2022 and has been held annually on Karuk ancestral territory. The Karuk TREX invites members of marginalized groups, with a focus on Indigenous women, to be trained in prescribed fire programs by local fire and cultural practitioners. The program is in collaboration with outside organizations like the Nature Conservancy, the Mid Klamath Watershed Council, and Fire Networks Women‐in‐Fire Prescribed Fire Training Exchanges (WTREX) to certify participants in varying aspects of prescribed fire. In this training program, participants are involved in all steps of the prescribed fire process, including landscape preparation, briefings, igniting, mop‐up (breaking up debris or stirring material to extinguish heat), and post‐fire monitoring (Figure 1).

Throughout the interviews, participants referred to the roles the Karuk people have in the landscape. Some participants explained that women were most likely responsible for managing basket materials, acorn groves, and medicinal plants for their families, communities, and ceremonies. They explained that women are needed in fire programs, offering a different perspective and holding valuable roles in sustaining the health and culture in their communities. This perspective is emphasized throughout the Karuk TREX program. The purpose of the program is to offer a safe and comfortable environment for Indigenous women to actively learn and build a relationship with fire, intending to reconnect participants with people and place. Leaf Hillman noted that with this program, “We can also empower basketweavers, ceremonial people, to manage the ceremonial areas with fire because they always have and they have continued to do it” (#5). To view a short video on the Karuk TREX program, see here: The Women They Are Carrying Fire – Pa'asik'tavaansas Kuniktáamvunatuh (2023).

Another part of Karuk's advocacy to restore cultural burning and preserve traditions is to partner with researchers in academia, the Forest Service, and other Tribal Nations. The Karuk are a leading Tribe in revitalizing cultural burning, sharing their knowledge of fire on the landscape, establishing research protocols, and continuously advocating for their inherent right to burn, a right never relinquished through treaties. They explained that for their people to survive on ancestral lands, there must be a shift in Tribal approaches to land management. For the Karuk, this shift requires the Karuk Peoples to not “blend in” anymore. Survival during European invasion and settlement led Karuk ancestors to hide by participating in cultural activities such as hunting and dancing at night, in order to continue to live as Karuk People. Leaf Hillman explained,

About 35 years ago we were fighting for dam removal and people were laughing at us, thinking that we weren't serious. It occurred to me that we can no longer blend in, we have to change strategies. Even strategies responsible for us existing, that strategy is no longer paying dividends, it is going to lead to our inevitable destruction. We have to stand up and be noticed. We had to have our voice amplified. Our people have never been good at that, we've been good at blending in. Which meant, don't be seen, don't be heard, don't oppose something, you don't stand up, you blend in because if somebody notices you, they're going to kill you. Time to change now; if we continue to blend in, we're going to get rubbed out (#5).

Participants added that there is a need for a community centered around Indigenous Fire Stewardship, a lesson passed down through oral storytelling. The participant explained a creation story, where fire was stolen from the people. To return fire, a community was created to steal it back. Hillman shared, “It took the mountain lion, coyote, the turtle, the rabbit, the bear, the taupe frog, and everyone else… The same thing is required now. It needs to be a coordinated effort from a lot of different entities to steal fire back because [the government] has taken fire from us” (#5). By working with outside researchers, the Karuk Tribe intends to build communities centered around Indigenous Fire Stewardship. These efforts will be critical for restoring Karuk ecocultural stewardship on post‐fire landscapes and more generally.

## 4 Discussion

Experiences shared by our participants demonstrate that settler colonialism persists through the external systems that attempt to control Karuk land and limit Karuk fire stewardship. The barriers participants described—limited authority, exclusion from decision‐making, and the need to constantly reeducate federal staff—are not just bureaucratic inefficiencies; they are the outcomes of a colonial system designed to undermine Indigenous governance and erase Indigenous knowledge. When federal agencies impose rigid protocols, ignore Karuk expertise, and/or cycle through staff without building lasting relationships, they are perpetuating a system that continues to dispossess the Tribe of land, power, and cultural continuity.

Participants made clear that these dynamics are not neutral; they reflect a refusal to prioritize Karuk sovereignty and the legitimacy of Indigenous fire knowledge. This is reflected in the funding challenges that impact the Karuk Tribe's ability to carry out fire stewardship practices. With only 900 acres of their ancestral territory designated as trust lands, many of the grants Karuk relies on are often awarded according to calculations on the basis of acreage or deliverables that do not align with their stewardship needs. As a result, the Tribe has to piece together funding from a combination of state, federal, and private sources, including CAL FIRE, the TFPA, and the RTRL. However, limited capacity within federal agencies often delays the process of acquiring burn approvals and implementing projects. Any funding allocated to the Tribe is put toward planning, implementation, and covering liability for intentional fire. At present, funding access remains uncertain because of potential shifts in federal priorities under the current presidential administration.

### 4.1 Connections to the Broader Literature

Existing literature discusses important differences in worldviews for land management/stewardship between settler colonial paradigms and Indigenous knowledge systems. Many Indigenous scholars have identified colonial perspectives as effectively separating humans from nature, or creating a man‐nature dichotomy, whereas Indigenous perspectives understand humans as a part of nature with a responsibility to steward the lands upon which they depend (Tom et al. 2023; Vinyeta and Lynn 2013; Whyte 2017, 2018). This difference is significant, as colonial worldviews have informed decades of land management policy and practices that have disrupted and currently neglect relationships between humans and nature. In forestry, this is showing up not just in the evolving understanding of the history of Indigenous fire stewardship (Hagmann et al. 2021) but also in the understanding of how Indigenous stewardship nurtured the development and maintenance of old‐growth forests (Eisenberg et al. 2024). Suppressing Indigenous stewardship and cultural burning traditions and then managing lands primarily for commodity production has led to land management paradigms that are detrimental to the land and people who live there.

In Western academic traditions predominant in natural resource management, relationships where humans access their material needs from a landscape and directly experience feedback within their community from the ecological systems they depend on are referred to as a coupled social‐ecological system. For the Karuk, however, the relationship is even deeper than this and requires an understanding of land stewardship in a context of reciprocity, that is, an ethic and lifeway that goes far beyond typical conceptualizations of social‐ecological systems. Kari K. Norgaard (2014a, 2014b); K. M. Norgaard (2019) has extensively discussed the Karuk People's reciprocal relationship with the land, where they are related to the natural world through spirit and have an inherent responsibility to support non‐human beings and the places that sustain them. This theme showed up consistently in our data when participants spoke about the significance of cultural burning on Karuk lands and within Karuk culture for their own political and food sovereignty, well‐being, religious freedom, and vitality. In contrast, by artificially separating or decoupling communities from the lands or resources upon which they depend and steward, settler‐colonial land management paradigms often lead to the degradation of places precisely because of the lack of feedback between the land and community. This creates cascading negative consequences for communities like the Karuk Tribe who live near lands designated for commodity production and depend on relationships with their homelands for survival. In our conversations with Karuk Tribal members and staff, people noted that their use of fire and communication of their stewardship responsibility were often misunderstood by federal agencies or seen to be at odds with federal land management objectives. Participants said that colonial views of land and resources as commodities have led to an ongoing resistance to acknowledging intentional fire as a means of wildfire mitigation and ecocultural revitalization; other scholars have documented this as well (Adams 2024; A. C. Christianson 2015; Hoffman et al. 2022; Layden et al. 2025).

A legacy of decoupled social‐ecological systems enforced by U.S. federal land managers has established and currently sustains negative impacts on Tribal Nations and their ancestral homelands. Participants consistently referred to bureaucratic barriers and the prevention of Indigenous fire stewardship as a modern form of settler colonialism. High‐intensity wildfire is also detrimental to resources vital to Indigenous lifeways and cultural practices, including nutritional foods, fibrous plants used in weaving and clothing, and wildlife populations significant to their relationships with non‐human beings and places. In these ways, the legacy of colonization continues to oppress Indigenous people who suffer from increased fire risk in their homelands and are legally and practically prevented from using fire as a means of protection and preservation of their communities and the resources they depend on.

Both Indigenous and non‐Indigenous scholars agree that protecting communities from climate‐related issues requires not only fuel mitigation but also reinstating, supporting, and amplifying Indigenous relationships with the land and their stewardship practices (Eisenberg et al. 2019; Whyte et al. 2023; Datta et al. 2024; Status of Tribes and Climate Change Working Group 2025). This is actively pursued and practiced throughout the Karuk community with implementations of cultural burning and coppicing on trust land alongside collaborations with researchers, the Karuk TREX program, and political education and advocacy through the *Good Fire I and II* reports. These efforts are intended to educate others on Karuk fire stewardship and support the Karuk people's connection to land (Clark et al. 2021; Clark, Tripp, et al. 2024; Varney et al. 2025). Achieving a more sustainable form of land stewardship involves harnessing a deep understanding of local ecology that can only be learned through generations of observation and practice. Melinda Adams and other Indigenous scholars have spoken to the growing recognition of Indigenous fire stewardship as a science to be considered alongside Western academic traditions (Adams 2024; Whyte 2020, 2017; K. M. Norgaard 2022). The Karuk Tribe is a leader in cultural burning advocacy and practice, producing numerous studies with outside researchers that highlight the social and ecological benefits of cultural burning.

This recognition has contributed to a broader movement for Indigenous data sovereignty for all Indigenous Communities whose knowledge and stewardship practices are increasingly being considered by Western academic traditions and land managers in the face of climate change. Many Indigenous scholars have stated that the recognition of Indigenous knowledges must be accompanied by a recognition of Indigenous rights, ownership, and authority in how their knowledges are applied and shared (Jennings et al. 2018; Hudson et al. 2023; David‐Chavez et al. 2024; Adams 2024). Karuk demonstrates this through their active efforts to have Karuk DNR employees on fires and planning teams, as well as through the use of the Practicing Pikyav protocol in research collaborations, which enforces Tribal ownership, co‐creation, and requires approval prior to publication. Previous scholarship has highlighted extractive practices in research, where non‐Indigenous institutions tend to appropriate Indigenous knowledges or data without Indigenous involvement or credit (Smith 2021; David‐Chavez and Gavin 2018). Indigenous scholars have developed frameworks to address research needs, such as the CARE Principles for Indigenous Data Governance (Collective benefit, Authority to control, Responsibility, and Ethics), which was developed as a complement to the FAIR (Findability, Accessibility, Interoperability, and Reusability) data principles (Jennings et al. 2023).

There are growing bodies of policy and practice recommendations, such as the Good Fire and the Status of Tribes and Climate Change (STACC) reports, that center Indigenous sovereignty in its fullest form, encompassing food, political, and data sovereignty (Whyte et al. 2023). These reports affirm our findings, emphasizing the need for Indigenous leadership and authority in cultural burning and fire management on ancestral lands. In our interviews, Karuk Tribal members were clear that Indigenous leadership and sovereignty, in data and practice, are essential for protecting cultural practices, sacred sites, stewarding land and resources, and ensuring the safety and self‐determination of their communities. Without it, efforts to care for the land risk being undermined, misunderstood, or made inaccessible by systems that have historically excluded Indigenous voices. We are beginning to see the state of California make legal strides toward supporting this shift and reflecting Tribal priorities, with recent legislation and policy frameworks acknowledging the legitimacy of cultural burning and its implementation with minimal federal oversight. However, these are just starting points, and additional work is needed.

In summary, our study contributes to broader bodies of literature that observe the negative environmental and social consequences of settler colonialism on both the land and Indigenous communities. These impacts jeopardize Indigenous cultures, disrupt sustainable stewardship practices, and harm the local environment. Research is increasingly acknowledging this disconnect and the need to revitalize Indigenous knowledges, particularly in the context of climate change and increasing wildfire frequency. Several other bodies of literature, including policy recommendations from reports such as the *Good Fire* and STACC, demonstrate how federal agencies can support Indigenous sovereignty and leadership in land stewardship to address these challenges. However, meaningful inclusion of Indigenous knowledges into federal systems requires the full acknowledgment of Indigenous rights and the active support of Indigenous leadership in the decision‐making process. Federal land and fire managers can take the important step of educating themselves about the Indigenous people and their fire stewardship traditions in the places where they work and actively seek out the venues to engage Indigenous ecocultural practitioners in land management on ancestral lands. Our research emphasizes that the revitalization of Karuk fire stewardship presents an opportunity for meaningful progress, especially in post‐fire landscapes, where traditional practices can play a pivotal role in post‐fire restoration. The success and longevity of these efforts remain contingent on the priorities of current and future U.S. political administrations, which ultimately determine how policies are implemented and where resources are allocated. A failure to support these practices perpetuates the legacy of colonialism and subsequent environmental degradation, undermining Indigenous sovereignty and excluding traditional ecological knowledge.

## 5 Conclusions

Our research aimed to identify the Karuk Tribe's post‐fire recovery priorities, explore management options to support those priorities, and examine governance systems impacting the Tribe. This research was limited to a small sample size of 13 interviews; we were not able to speak with everyone we had intended to because of scheduling conflicts and limited availability. Our fieldwork was time‐constrained by graduate school and grant timelines, with only seven days total spent in Karuk aboriginal territory to conduct in‐person interviews and a year to analyze data and write up findings. Although this was intentional for the scope of our research, further studies would be needed to understand how agencies like the U.S. Forest Service, particularly within the Klamath and Six Rivers National Forests, view post‐fire management and Tribal collaboration. Additionally, our findings should not be generalized to all Tribal Nations or even all Karuk Tribal members, as each participant offered a unique and individual perspective. Similarly, the study does not fully capture the cultural context, ceremonial knowledge, or stewardship practices that exist within Karuk fire traditions; knowledge and practice are deeply rooted in Karuk culture and traditions that cannot be fully shared or understood through limited interviews or outside perspectives.

Participants consistently spoke about the ongoing social challenges following the Slater Fire, including a severe housing shortage and how it is resulting in a lack of essential workers, like teachers, dentists, and healthcare providers, in their communities. They said these issues were limiting the community's ability to access proper and affordable health care, education, housing, and food. More research is needed to assess the broader social impacts of the Slater Fire on Tribal communities. Finally, we suggest learning more about the Karuk Women's TREX program, its significance, the roles it plays in cultural revitalization, and how it is connecting with other Indigenous communities and global Indigenous fire movements. The program is a powerful example of how Indigenous fire stewardship can center and support genders, creating space for women and Two‐Spirit people to lead, learn, and reclaim their traditional roles in fire practice and governance.

Looking ahead, future research should be designed with an awareness of the structural limitations and ethical responsibilities that shape work with Indigenous communities. This includes recognizing the complexities of scheduling, as the availability of potential participants may be limited because of seasonal ceremonies, traditional practices, or other circumstances that can restrict opportunities for in‐person interactions, which are often preferred. Future research should recognize that seasonal availability can further influence the knowledge participants share, as certain information may be more accessible or relevant during specific times of the year. Additionally, researchers could further investigate other complexities in fire management, such as funding sources that support land and fire management projects, the constraints associated with those funds, and the deliverables or timelines they may impose. Furthering education on foundational understandings of Indigenous data sovereignty also remains important. Researchers, institutions, and federal partners must recognize that Indigenous Peoples and their knowledge systems are not monolithic. Researchers must approach their work in a way that respects Indigenous sovereignty and design research that allows for and prioritizes Indigenous ownership, authorship, and control over how their knowledge and cultural information is used or shared. This also includes understanding how policies are shaped, who benefits from them, and who is excluded.

There are differences in management styles and worldviews between federal agencies and Indigenous communities, many of which are shaped by histories of colonialism, sexism, racism, and the exclusion of non‐dominant perspectives from formal systems, such as education, training, and governance. Future researchers should not only be aware of this context but also be willing to ask questions and explore how these dynamics show up in decision‐making spaces, collaborations, and everyday interactions between diverse rights‐holders. This includes understanding the deep relationship between society and the environment. In the context of this work, intense wildfires do not just damage landscapes; they reduce the resources that Indigenous communities rely on to recover, often increasing their dependence on government support. That support is frequently tied up in bureaucratic red tape, which compounds trauma and slows down recovery. The more communities of practice educate themselves on these interrelated issues, the better equipped they are to support Tribal Nations like the Karuk Tribe in restoring their relationships to land, fire, and culture.

Despite ongoing bureaucratic barriers, the Karuk Tribe is asserting its inherent right to steward fire and reclaim relationships with its ancestral land. Participants emphasized that cultural burning is essential not only for ecological healing but for the survival of Karuk culture, governance, and identity. This advocacy is significant as fire stewardship is not just a practice; it is how Karuk ancestors protected their lands, sustained their communities, and ensured their survival. The Karuk people are here today because their ancestors fought and survived to maintain these practices in the face of colonial violence and erasure. Today's efforts to revitalize cultural burning are a continuation of that resistance. These efforts are deeply rooted in the assertion of Karuk sovereignty, the right to burn without external interference, criminalization, or delay (Clark et al. 2021; Clark, Tripp, et al. 2024).

Through advocacy, knowledge‐sharing, and leadership, the Karuk Tribe is challenging the bureaucratic systems that continue to limit Indigenous fire stewardship. Trainings like the Karuk Indigenous Women‐in‐Fire TREX are not just workforce development; they are about reconnecting people with each other, ancestral knowledge, and the land. This work honors the legacy of Karuk ancestors who burned and resisted. By sharing their story and knowledge, they ensure that future generations can do the same.

## Author Contributions

**Caitlyn Cruz:** data curation (equal), formal analysis (lead), investigation (lead), methodology (equal), writing – original draft (lead), writing – review and editing (equal). **Kathy McCovey:** investigation (equal), project administration (supporting), supervision (supporting), validation (lead), writing – original draft (supporting), writing – review and editing (supporting). **Gregory Russell:** conceptualization (equal), data curation (equal), formal analysis (supporting), investigation (supporting), methodology (supporting), project administration (supporting), supervision (supporting), writing – original draft (supporting), writing – review and editing (supporting). **Dominique David‐Chavez:** conceptualization (equal), project administration (supporting), supervision (equal), writing – review and editing (supporting). **Lindsey Schneider:** conceptualization (equal), project administration (supporting), supervision (supporting), writing – review and editing (supporting). **Courtney Schultz:** conceptualization (lead), data curation (lead), formal analysis (supporting), funding acquisition (lead), investigation (lead), methodology (lead), project administration (lead), resources (lead), supervision (lead), writing – original draft (supporting), writing – review and editing (lead).

## Funding

Funding for this project was awarded by the DOI‐USGS Southwest Climate Adaptation Science Center and supported by graduate student opportunities within the Department of Forest and Rangeland Stewardship at Colorado State University.

## Conflicts of Interest

The authors declare no conflicts of interest.

## Data Availability

All of our data are considered confidential human subjects data that are not fully deidentifiable. Colorado State University's Institutional Review Board does not allow them to be shared except with other researchers on our team with their own IRB approval. We have made the editorial board of Ecology and Evolution aware of this contingency, and they allowed the review process for our submission to move forward.
